# Affective forecasting dynamics as an early intervention target in depression: evidence from ecological monitoring and temporal network analysis

**DOI:** 10.3389/fpsyt.2025.1739976

**Published:** 2026-01-12

**Authors:** Zhuoya Yang, Xuanang Liu, Yating Wang, Fenghua Li, Yixiao Fu, Zhengzhi Feng, Lei Xia, Chunmeng Shi

**Affiliations:** 1 Department of Basic Psychology, School of Medical Psychology, Army Medical University, Chongqing, China; 2 School of Medical Psychology, Army Medical University, Chongqing, China; 3 Key Lab of Mental Health, Institute of Psychology, Chinese Academy of Sciences, Beijing, China; 4 School of Labor and Human Resources, Renmin University of China, Beijing, China; 5 Department of Psychiatry, The First Affiliated Hospital of Chongqing Medical University, Chongqing, China; 6 Experimental Research Center for Medical and Psychological Science, School of Medical Psychology, Army Medical University, Chongqing, China; 7 Institute of Rocket Force Medicine, State Key Laboratory of Trauma, Burns and Combined Injury, Army Medical University, Chongqing, China

**Keywords:** affective forecasting, depression, experience sampling method, heart rate variability, temporal network analysis

## Abstract

**Background:**

Deficits in affective forecasting are closely associated with the development and maintenance of depression. While previous research has shown that emotional fluctuations and future thinking are prevalent in daily life, little is known about the psycho-physiological mechanisms of affective forecasting deficits in relation to specific daily events in depression. Methods: Dysphoric (N = 40) and non-dysphoric (N = 60) individuals completed assessments of their anticipatory emotions, experienced emotions, and consummatory emotions for specific daily events 3 times a day for consecutive 14 days. Heart rate variability (HRV) data was collected using customized smartwatches. Temporal network analysis was used to estimate time-lagged associations among anticipatory, experienced and consummatory emotions.

**Results:**

Dysphoric individuals reported significantly lower levels of anticipatory, experienced, and consummatory valence compared to non-dysphoric individuals. Furthermore, distinct patterns emerged in the temporal networks of anticipatory, experienced, and consummatory emotions between the dysphoric and non-dysphoric groups. Network density was considerably higher in dysphoric individuals than in non-dysphoric individuals. In addition, HRV was predictive of anticipatory valence across all participants. Moreover, the dynamic associations between anticipatory and experienced emotions predicted subsequent depression, even after accounting for baseline depressive symptoms.

**Conclusion:**

Our findings reveal provide novel insights into the psycho-physiological mechanisms of affective forecasting deficits in depression, with several clinical implications: (1) dysfunctional affective forecasting dynamics may serve as salient early warning signs and sensitive predictors of depression; and (2) improving the flexibility of affective forecasting may be a promising target for addressing depression.

## Introduction

The predictive processing model posits that the human brain functions not merely as a stimulus-driven machine; rather, it actively anticipates and predicts information in advance (1). This ability is crucial for decision making and future coping (2). Consistent with this framework, the influential cognitive triad suggests that negative views of future constitute a fundamental aspect of depression (3). Substantial evidence supports the existence of negative biases in affective forecasting (e.g., blunted positive emotion prediction bias; 4) among both individuals with subclinical depression and patients with major depressive disorders (MDD). These biases are closely linked to the severity of depressive symptoms (5). Consequently, they may lead to anhedonia/amotivation (6), hopelessness (5), and even suicide (7). Thus, biased affective forecasting may serve as a salient early warning sign of depression and a pivotal target for early intervention.

In the literature, affective forecasting has been primarily measured by anticipatory emotions (predictions of emotional experiences during future events), experienced emotions (emotional experiences in relation to a predicted event occurring) and prediction bias (comparisons between antipatory and experienced emotions; 4). Previous experimental studies have consistently found blunted anticipatory pleasure and a lack of optimistic prediction bias in both individuals with subclinical depression and MDD patients (4, 8). However, whether anticipatory negative emotions enhance or not in depressed people remains a matter of debate (9). One possible explanation for these conflicting findings may lie in methodological limitations. Future thinking and anticipatory emotions have been found to be highly personally relevant and frequently occur in daily life (10). The majority of prior studies applied standardized laboratory tasks and measured only one or two time points. Therefore, they may fail to accurately capture the dynamic nature of anticipatory emotions in everyday contexts.

The experience sampling method (ESM) is a technique involving repeated sampling of real-time cognition and emotion in daily life (11). Its advantages include high ecological validity and minimal recall bias. The ESM has been regarded as a “gold standard” to efficiently capture fluctuations in emotional experience (12). Several ESM studies have investigated affective forecasting in depression (4, 13, 14). A stronger negative prediction bias and a blunt positive prediction bias towards general affect over the upcoming week has been found in dysphoric individuals and MDD patients (15, 16). However, these studies failed to capture fluctuations in anticipatory emotions towards daily events, which may offer specific targets for future interventions. Several studies have suggested that both individuals with MDD (14) and those with dysphoria exhibit blunted anticipatory pleasure for daily. Nevertheless, these studies have only measured the intensity of pleasure experiences. To provide a more comprehensive understanding toward affective forecasting deficits in depression, the present study examined two dimensions—valence (positive, negative) and arousal (low, high)—of both anticipatory emotions and experienced emotions related to specific daily events.

Moreover, the temporal dynamics of affective forecasting are not well-understood. Most prior studies have assessed forecasting accuracy at the between-subject level (4). Nevertheless, such a focus overlooks within-subject fluctuations that may be crucial for detecting subtle changes in subclinical or clinical samples. Li (13) found predictive associations between anticipatory pleasure and subsequent consummatory pleasure (pleasure experience during current events) in both dysphoric individuals and controls using multilevel modeling. However, it is important to note that anticipatory and consummatory pleasure may not necessarily reflect hedonic responses to identical events within this study.

Evidence indicates that when the occurrence of the predicted event either precedes or follows the current event, consummatory emotions may serve as significant contextual factors impacting anticipatory and experienced emotions (17, 18). A critical distinction was made between consummatory emotions and experienced emotions. Consummatory emotions refer to the in-the-moment affective state individuals experience while engaged in any current activity. In contrast, experienced emotions specifically capture the affective response to a particular event that was previously anticipated. This distinction is crucial for isolating affective forecasting (anticipatory vs. experienced emotions for the same event) from current mood states (consummatory emotions). In present study, ESM was combined with temporal network analysis to provide a more accurate estimation of dynamic interactions between anticipatory emotions, experienced emotions and consummatory emotions in dysphoric and non-dysphoric individuals. On the other hand, prior research has inadequately explored the longitudinal relationship between affective forecasting and depressive symptoms. In this study, we further investigated whether these daily emotions and the temporal network could predict subsequent levels of depression. Our findings might shed a light on the mechanism of affective forecasting deficits in depression and facilitate future early interventions.

In addition, although heart rate variability (HRV) has been shown to be closely related to emotional experience (19, 20), the relationship between HRV and affective forecasting remains largely unexplored. Therefore, another objective of our study was to examine associations between fluctuations in HRV and the dynamics of anticipatory emotions in daily life. This investigation could contribute to better understanding of the physiological underpinnings of affective forecasting deficits in depression and potentially identify biomarkers for early intervention.

The present study aimed to examine the dynamics of affective forecasting by employing ESM in conjunction with temporal network analysis among dysphoric individuals. Investigating these at-risk individuals may elucidate the mechanisms underlying the future development of MDD episodes. This pre-onset research design also excludes the confounding influences of factors such as medication and episode frequency. A further objective of this study was to investigate the predictive impact of anticipatory emotion dynamics on subsequent changes in depressive symptoms. Additionally, we preliminarily explored the physiological mechanisms underlying affective forecasting deficits by assessing correlations between anticipatory emotions and HRV. It was hypothesized that dysphoric individuals would report more negative anticipatory and experienced emotions compared to non-dysphoric individuals. Furthermore, it was anticipated that dysphoric individuals would exhibit altered affective forecasting dynamics, which would serve as significant predictors of depressive symptoms at follow-up. Our findings might provide novel insights into the mechanisms of affective forecasting deficits in depression and boost early detection and intervention.

## Materials and methods

### Participants

First, we invited college students (N = 403) from Chongqing, China complete the BDI and the MDQ via online links. The BDI-II was adopted to assess depressive symptoms (cutoff: < 14 minimal depression; 14–19 mild depression; 20–28 moderate depression; 28–63 severe depression; 21, 22). While the Mood Disorder Questionnaire (MDQ) was widely used to screen for bipolar disorder (23, 24). Participants who scored 7 or higher on the MDQ were excluded (25). Then participants scoring 20 or above on the BDI were classified into the dysphoric group. In contrast, those scoring below 14 constituted the control group. This classification criterion aligns with previous studies that utilized college student samples (13, 26). Potential participants were further screened by phone according to the following exclusion criteria: a history of brain injury, substance use or psychiatric disorders. Finally, 40 dysphoric individuals (age: 20.19 ± 1.45; gender: 84.91% female) and 60 controls (age: 19.76 ± 1.58; gender: 86.84% female) were included in the current study (**Table 1**). This study was approved by the Ethics Committee of the Army Medical University. Written informed consent was obtained from participants at the beginning of the baseline assessment.

**Table 1:** Sample characteristics.

Sample characteristics	Control group (N = 53)	Dysphoric group (N = 38)	t/χ^2^	p
	(*M* ± *SD*)	(*M* ± *SD*)		
Age	20.19 ± 1.45	19.76 ± 1.58	1.29	0.20
Gender (M/F)	8/45	5/33	0.07	0.80
BDI_T1	2.74 ± 3.75	24.76 ± 4.15	-26.42	<0.001
MDQ	2.68 ± 2.13	3.16 ± 1.35	-1.31	0.19
TEPS_total	87.53 ± 12.38	81.53 ± 18.88	1.83	0.07
TEPS_ANTI	39.81 ± 5.90	37.19 ± 6.87	1.94	0.06
TEPS_CON	47.72 ± 7.55	46.54 ± 7.51	0.73	0.47
NBPS	42.70 ± 4.79	41.73 ± 5.14	0.92	0.36
PHQ_T1	3.09 ± 3.73	11.35 ± 3.66	-10.41	<0.001
PHQ_T2	4.11 ± 4.06	11.57 ± 4.89	-7.63	<0.001
BDI_T2	10.81 ± 11.04	14.76 ± 11.33	-1.67	0.09

BDI, Beck Depression Inventory; MDQ, The Mood Disorder Questionnaire; TEPS, The Temporal Experience of Pleasure Scale; ANTI, Anticipatory pleasure; CON, Consummatory pleasure; NBPS, The Negative Bias in Prospection Scale; PHQ, The Patient Health Questionnaire-9; T1, baseline measurement point; T2, follow-up measurement point.

We used the pilot data (10 participants per group) to estimate the sample size. A power analysis was conducted using the Monte Carlo simulation (the SIMR 1.0.2 package; 27) to determine the sample size for detecting significant between-group differences, which was a major focus of this study. The power reached 0.80 when the sample size was 89. Therefore, this study ultimately included 100 participants.

### Baseline and following-up assessment

Participants completed several self-report scales within one week before the beginning of the ESM procedure. The Patient Health Questionnaire-9 (PHQ-9; 28)was used to measure depressive symptoms before and after the ESM procedure. Frequencies of depressive symptoms were rated on a 4-point Likert scale from 0 (“not at all”) to 3 (“nearly every day”). The Chinese version of the PHQ has good reliability and validity (29). The Temporal Experience of Pleasure Scale (TEPS; 30, 31) was adopted to assess trait anticipatory and consummatory pleasure. It is scored on a 6-point Likert scale from 1(“very false for me”) to 6 (“very true for me”), with higher scores indicating greater hedonic capacity. The Chinese version of the TEPS has been shown to possess good internal consistency (Cronbach’sα = 0.759). The Negative Bias in Prospection Scale (NBPS; 32) was a 14-item self-report instrument used to measure prospection bias. It consists of three factors, namely increased negativity, reduced positivity, and overgeneralization. The NBPS is rated on a 5-point Likert scale (1= totally disagree to 5 = totally agree). Higher scores indicate higher levels of negative bias in prospection. The NBPS has demonstrated to have good psychometric properties (Cronbach’sα = 0.86; 32). Within one week after the ESM procedure, all participants were administered the BDI and the PHQ again.

### ESM procedure

An online link containing the set of ESM assessments was sent to each participant’s smart phone 3 times a day (first 07:30 a.m.- 08:30 a.m., second 1:40 p.m.- 2:40 p.m., and third 8:00 p.m.- 9:00 p.m.) for 14 consecutive days. This schedule was implemented for several important reasons: (1) to capture affective experiences across key periods of the day; (2) to enhance participant compliance by aligning with daily routines; and (3) to ensure a comparable distribution of data points for the temporal network analysis (33). This yielded a maximum of 42 events per participants. They were asked to complete the ESM assessments as soon as they received the message. Prompts were sent if no response was given for 1 hour. And the data were considered missing if no response was given for more than 3 hours. Two participants from the control group dropped out at the first day due to scheduling problems.

**Figure 1:**
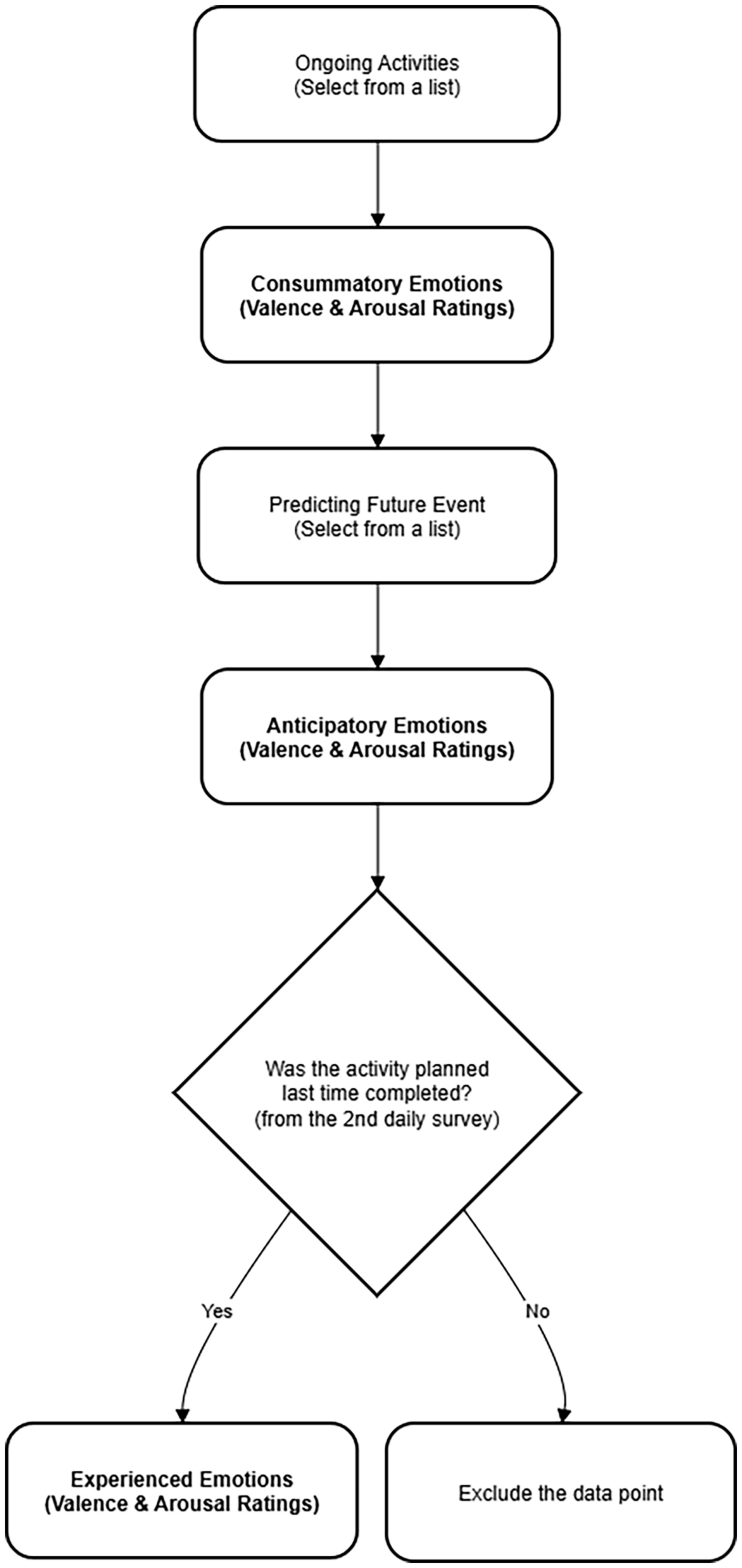
A flowchart schematically depicting the temporal sequence of a typical ESM survey.

At the beginning of each survey, participants indicated their ongoing activity by selecting from a list of options (work/study, sleep, eating, daydreaming, sports, cleaning, playing games, watching videos, chatting online, shopping, watching movie, going on a date, group activities, others). Then they were asked to report consummatory emotions (question: “what’s your feeling at this moment?”; two ratings: valence from -7 “very unpleasant” to 7 “very pleasant”; arousal from -7 “very calm” to 7 “very excited”). They were also asked to select what activity they would be engaged in next and predict emotion experience during the future activity (question: “How do you think you will feel?”; anticipatory emotions: both valence and arousal). To assess experienced emotions, participants were instructed to rate two additional items from the second ESM survey: (1) “Have the activity planned last time been completed?” (1 “yes”, 0 “no”); if the response was “Yes”, they continued to answer the second question “What was your feeling during that activity?” (both valence and arousal). If the response was “No” to the first question, this measurement point was excluded in the following analysis (**Figure 1**). In this way, experienced emotions would not overlap with consummatory emotions. This design explicitly ties experienced emotions to a prediction-experience loop, whereas consummatory emotions represent current mood states. **Figure 1** schematically depicts the temporal sequence of the ESM measurements. For quality control, an attention item (e.g., “please choose ‘3’ for the answer.”) was randomly presented in every ESM survey. The data was deleted if the answer was wrong. Finally, seven participants were removed from the final analysis because their valid response rates were below 50%. Fifty-three controls (valid measurement points = 40.96 ± 1.72) and 38 dysphoric individuals (valid measurement points = 40.45 ± 1.93) remained in the final sample pool. Records containing missing values were deleted in the following analyses.

### Physiological recording and analysis

R-R intervals (RRI) were collected using the same customized smartwatches as those in Li et al. (34)’s study. Huawei band 8 was adopted, and the sampling frequency of PPG was set at 25 Hz (34). Each of participants was asked to wear the smartwatch on the non-dominant hand side. RRI data was collected continuously for 14 days during the whole ESM procedure and was sent out to the cloud server via a smartphone application as soon as possible. To control the influence of behavioral factors on the HRV data, the ESM assessments comprised also several items, such as the body posture, social activity, eating, drinking and physical activity (see **Supplementary Table S1**). The preprocessing pipeline for HRV analysis relied on built-in algorithms of the Huawei smartwatches (34, 35). They are designed to assess signal quality for reliable heart rate derivation. Specifically, these algorithms involve peak detection, physiological feasibility rules (e.g., heart rate range checks), and adaptive template matching to compute correlation coefficients for quality assessment.

For data analysis, 30 min of continuous recording prior to the ESM assessments were parsed. And the R-R Intervals (RRI) data were mapped on to the time periods just prior to the ESM surveys. This 30-minute window was applied to mitigate random noise caused by factors like posture changes, eating or movement, thereby yielding more stable signals (36). The root means square successive difference of interbeat intervals (RMSSD) was calculated for every 300-second period and then averaged across the whole 30-minute segment. The RMSSD is a robust index reflecting vagal control on the heart. And it is less susceptible to movement and respiratory influences.

### Data analysis

First, SPSS 26.0 (Chicago, IL, USA) was used to calculate group differences on demographic information and baseline assessments. The ESM data were analyzed in R 4.4.2 (37). We employed multilevel modeling and temporal network analysis. Multilevel modeling accounts for the nested structure of the ESM data (e.g., repeated measures within individuals). Whereas temporal network analysis estimates directed, lagged associations between variables across time points. These analyses allow for a robust investigation of affective forecasting processes at both intra-individual and inter-individual levels.

Multilevel models were built with within-person daily surveys as the Level 1 variables, and between-person characteristics as Level 2 variables. We estimate separate models for consummatory, anticipatory and experienced emotion. All Level 1 variables were person-mean centered, and intercepts and slopes of Level 1 were modeled as random effects. To examine whether depression status predicted differences in consummatory, anticipatory and experienced emotion across daily surveys, the within-person variables were regressed on group (i.e., 0 = non-dysphoric individuals; 1 = dysphoric individuals). To investigate the relationship between RMSSD and anticipatory emotion, we regressed anticipatory valence and anticipatory arousal on RMSSD, with behavioral factors and group as controlling variables.

To examine the dynamic associations between anticipatory, consummatory and experienced emotion, temporal networks were estimated using the mlVAR package in R. Each temporal network is a directed network of regression coefficients indicating lagged correlations between nodes from time t-1 to time t, controlling for all other variables at time t-1. In the current study, time t - 1 and time t refer to two consecutive ESM surveys within the same day. Overnight intervals were excluded (33) for several reasons: (1) to focus on intradaily dynamics during waking hours; (2) adhering to the assumptions of the multilevel vector autoregressive (mlVAR) model for accurate parameter estimation; and (3) ruling out possible confounding variables. Six nodes were included: anticipatory valence, anticipatory arousal, consummatory valence, consummatory arousal, experienced valence, and experienced arousal. The temporal emotion dynamics were estimated within a multilevel modeling framework, allowing for random, person-specific regressive effects. Therefore, both group (fixed) and individual (random) effects were estimated. Orthogonalization is recommended in mlVAR modeling when the number of variables reaches six or more (38, 39).

We visualized temporal networks using the R package graph. In addition, the network density was calculated by averaging over the absolute values of all edges in a network. Group difference of network density was calculated using the independent t test. To examine group differences in affective forecasting, independent t tests were conducted to compare values of edges between anticipatory and experienced emotions. The Bonferroni method was adopted to correct multiple comparisons. Sensitivity analyses incorporating overnight lags or adopting the non-orthogonal method were conducted to assess the robustness of the temporal network findings. The results demonstrated network structures analogous to the original (**Supplementary Figures S1**, **S2**). Moreover, the dysphoric group consistently exhibited greater network density than controls (including overnight lags:t(89) = -8.97, p < 0.001; non-orthogonal method: t(89) = -2.62, p= 0.01).

Finally, to investigate whether daily emotion and RMSSD predict later depressive symptoms, subsequent depression level was regressed on the six emotion variables and RMSSD, controlling for behavioral factors and baseline depression. Moreover, to further examine whether temporal dynamics of affective forecasting predict subsequent depression, we also regressed subsequent depression level on edges between anticipatory and experienced emotions, controlling for baseline depression. Bidirectional stepwise regression was performed to select the optimal linear model by iteratively adding/removing variables. The Akaike Information Criterion (AIC) was used as the selection metric.

## Results

The two groups did not differ in age, gender and ratings on the MDQ. The baseline BDI (*t* (89) = -26.42, *p* < 0.001) and PHQ scores (*t* (89) = -10.41, *p* < 0.001) were higher in dysphoric individuals than in controls. There were no significant group differences on the TEPS (total: *t* (89) = 1.83, *p* = 0.07; anticipatory pleasure subscale: *t* (89) = 1.94, *p* = 0.06; consummatory pleasure subscale: *t* (89) = 0.73, *p* = 0.47) and the NBPS (*t* (89) = 0.92, *p* = 0.36), which may suggest that dysphoric individuals exhibit similar levels of trait anticipatory or consummatory pleasure and prospection bias as controls. Results were listed in **Table 1**.

### ESM estimates and HRV measures

Dysphoric individuals reported significantly lower levels of consummatory valence (*B* = -0.43, *SE* = 0.10, *p* < 0.001), anticipatory valence (*B* = -0.48, *SE* = 0.11, *p* < 0.001) and experienced valence (*B* = -0.43, *SE* = 0.12, *p* < 0.001) than controls. And individuals with dysphoria experienced a higher level of consummatory arousal than controls (*B* = 0.27, *SE* = 0.13, *p* = 0.04). No group difference was observed in anticipatory arousal (*p* = 0.19) and experienced arousal (*p* = 0.58) between the two groups. In addition, lower RMSSD was found in the dysphoric group, compared with the control group (*B* = -0.16, *SE* = 0.07, *p* = 0.02; **Table 2**).

**Table 2:** Estimates of anticipatory, experienced and consummatory emotions in dysphoric individuals compared with controls.

Outcome variable	*B*	*SE*	*p*
Anticipatory valence	-0.48	0.11	< 0.001
Anticipatory arousal	0.19	0.14	0.19
Experienced valence	-0.43	0.12	< 0.001
Experienced arousal	0.07	0.12	0.58
Consummatory valence	-0.43	0.10	< 0.001
Consummatory arousal	0.27	0.13	0.04

As for the relationship between HRV and anticipatory emotion, we found that RMSSD was a significant predictor of anticipatory valence (*B* = 0.002, *SE* = 0.001, *p* = 0.04), after controlling for the dysphoric status (i.e., group) and behavioral factors which might affect HRV data.

### The temporal network

**Figure 2:**
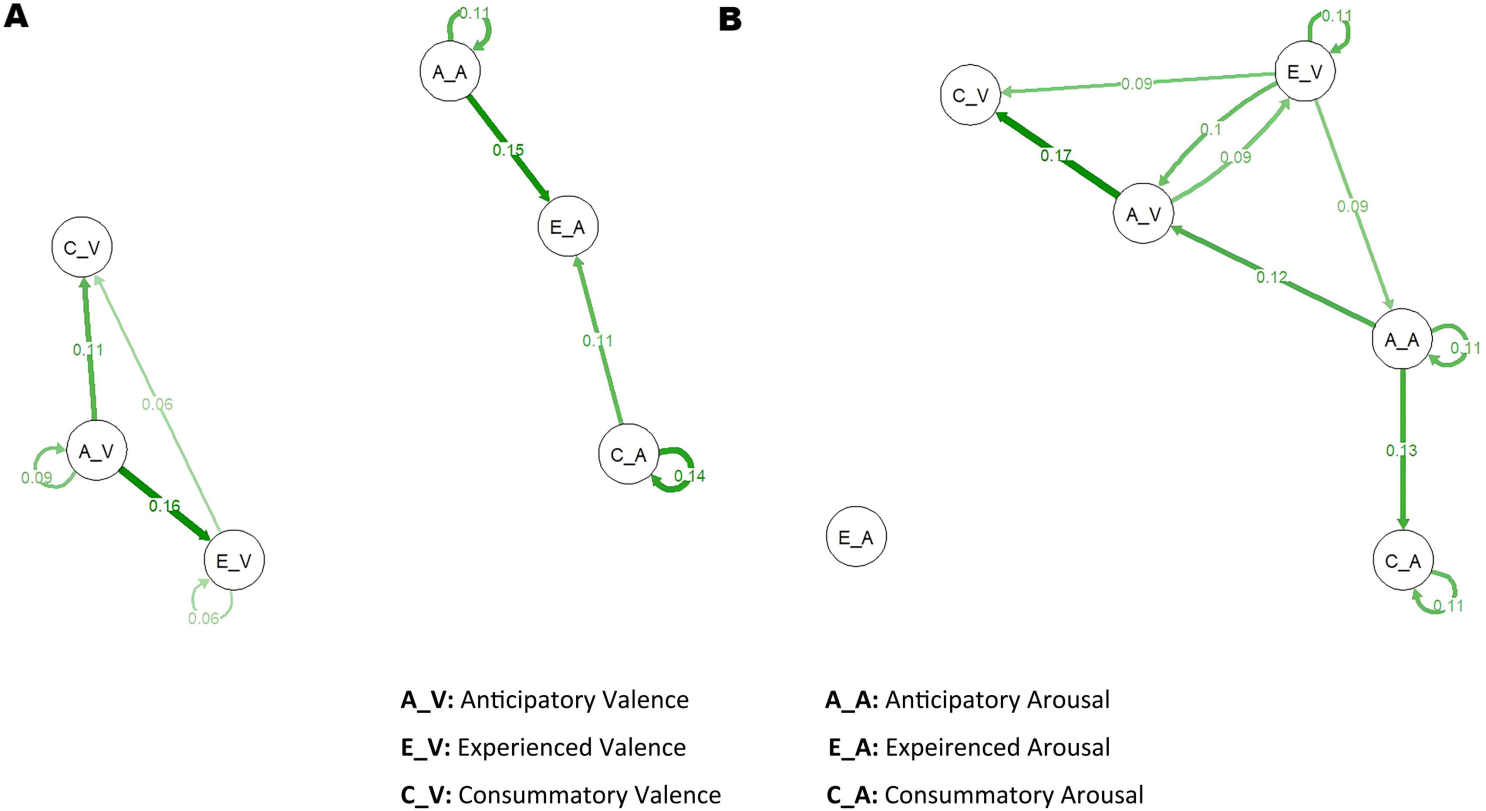
Temporal networks of anticipatory, experienced and consummatory emotions in non-dysphoric **(A)** and dysphoric **(B)** individuals. The green lines represent positive partial correlations. Thicker edges represent stronger associations between nodes. Only significant edges were retained (p < 0.05).

First, the average network in each group was modeled and visualized (**Figure 2**). Average networks reflect general patterns of temporal associations between the emotion variables. Green lines represent positive connections. The thickness of the line indicates the strength of the correlation. As shown in **Figure 2**, the valence and arousal seemed to be two separated subsystems in the control group (**Figure 2A**). While in dysphoric individuals, valence and arousal interacted with each other (**Figure 2B**). With regard to the anticipatory emotions, anticipatory valence could positively predict experienced valence (dysphoria: *B* = 0.09, *p* = 0.035; control: *B* = 0.16, *p* < 0.001) and consummatory valence (dysphoria:*B* = 0.17, *p* < 0.001; control:*B* = 0.11, *p* = 0.015) in the next measurement point in both groups. However, anticipatory arousal predicted experienced arousal only in the control group (*B* = 0.15, *p* < 0.001). There was no significant predictor for experienced arousal in dysphoric individuals.

The independent t test suggested network density was significantly greater in dysphoric individuals than in controls (dysphoria: 0.07 ± 0.01, control: 0.06 ± 0.01, *t*(89) = -3.34, *p* = 0.001). Moreover, group differences were observed in dynamic interactions between anticipatory emotion and experienced emotions (**Supplementary Table S2**). Specifically, the temporal link from anticipatory valence to experienced valence was significantly weaker in the dysphoric group than controls (*t*(78.92) = 3.27, *p* = 0.002). While the correlation from experienced valence to anticipatory valence was stronger in dysphoric individuals than in controls (*t*(89) = -7.75, *p* < 0.001). Temporal associations between valence and arousal were predominantly stronger in the dysphoric group than in the control group (e.g., anticipatory valence to anticipatory arousal, *t*(45.81) = -4.16, *p* < 0.001; experienced valence to anticipatory arousal, *t*(85.41) = -6.50, *p* < 0.001; anticipatory arousal to anticipatory valence, *t*(37) = -8.05, *p* < 0.001).

### Prediction of subsequent depression

Regression analysis suggested that neither the RMSSD nor daily emotion variables could significantly predict subsequent depression, after controlling for baseline depressive symptoms and behavioral factors (all ps’ > 0.05).

However, we found that subsequent depression could be predicted by dynamic associations between anticipatory emotions and experienced emotions, controlling for baseline depressive symptoms. Autoregressions of anticipatory valence (B = 3.86, SE = 0.98, p < 0.001) and experienced valence (B = 1.85, SE = 0.81, p = 0.03), as well as cross-lagged correlation from anticipatory valence to anticipatory arousal could positively predict subsequent depression (B = 3.04, SE = 0.09, p = 0.001). Cross-lagged link from anticipatory arousal to experienced valence could negatively predict subsequent depression (B = -2.96, SE = 0.79, p < 0.001).

## Discussion

Deficits in affective forecasting have been closely linked to the development and maintenance of depression (5). Previous research has indicated that emotions fluctuate and future-oriented thinking frequently occurs in daily life (10). However, less is known about the temporal dynamics of anticipatory and experienced emotions in daily events among individuals with depression. In the present study, we employed ESM and temporal network analysis to examine affective forecasting in both dysphoric and non-dysphoric individuals. Our findings revealed that dysphoric individuals reported lower levels of anticipatory, experienced, and consummatory valence compared to controls. Furthermore, distinct patterns in the temporal networks of anticipatory, experienced, and consummatory emotions were observed between the dysphoric and control groups. The results indicated that network density was significantly greater in dysphoric individuals than in controls. Moreover, dynamic associations between anticipatory and experienced emotions were found to predict subsequent depression, even after controlling for baseline depressive symptoms. In addition, we found HRV was significantly correlated with anticipatory valence. These findings enhance the understanding of daily changes in affective forecasting among individuals with depression and provide novel targets for early detection and intervention.

Prior research did not differentiate consummatory emotions and experienced emotion. In current study, we further identified characteristics of the two components of affective forecasting (anticipatory and experienced emotions) and the important influencing factor (consummatory emotions) in depression. We found reduced levels of anticipatory, experienced, and consummatory valence in dysphoric individuals compared to non-dysphoric individuals. Previous studies have similarly reported diminished anticipatory and consummatory pleasure in MDD patients (14) and dysphoric individuals (13). It is also in line with the finding that MDD patients have been observed to exhibit more negative daily affect relative to healthy controls (15). These findings suggest that dysphoria is linked to heightened negative emotions during both anticipatory, experiential and current experience. On the other hand, our results indicated that dysphoric individuals reported a higher level of consummatory arousal than controls. This observation is consistent with earlier studies showing that depressed people experienced increased arousal (40, 41). One possible explanation is that depressed people may perceive their environment as more stressful than healthy controls, thereby intensifying their physiological reaction (41).

Moreover, we marks the first attempt to explore the relationship between anticipatory emotions and HRV. Firstly, we found decreased RMSSD in dysphoria, consistent with prior studies on both at-risk individuals (42) and MDD patients (43, 44). Reduced RMSSD has been suggested to involve in maladaptive emotion processing and impaired motivation (45). Moreover, our findings suggested that the RMSSD was a significant predictor of anticipatory valence across all participants. This may reflect that vagally-mediated cardiac flexibility is closely related to generation of more positive anticipatory emotions. One possible explanation is that, optimal vagal tone (higher RMSSD), may facilitate prefrontal cortex inhibition of amygdala reactivity (46), thereby enabling more adaptive affective forecasting. While further investigation is necessary, our results indicated a link between dysfunctional HRV and affective forecasting deficits in dysphoria. This positions RMSSD as a promising biomarker for monitoring at-risk individuals, where lower RMSSD may signal maladaptive anticipatory processes. Moreover, It could be targeted with interventions like vagal nerve stimulation or respiration biofeedback aimed at improving vagal tone.

One of our important findings is that distinct patterns in the temporal network between two groups. Firstly, the overall network density was greater in dysphoric individuals than in controls. Previous studies examining in-the-moment emotion experience have suggested denser emotion networks in both dysphoria and MDD patients (47), which may reflect emotion inflexibility in these populations. The current study extends prior findings by highlighting the inflexibility of affective forecasting dynamics in dysphoria. Secondly, we identified a closer relationship between valence and arousal in dysphoric individuals compared to controls, consistent with previous research indicating that dysfunctional arousal significantly impacts valence in depressive disorders (48). Previous studies on affective forecasting have concentrated on biased valence associated with psychopathology (4). Our findings highlighted increased dynamic interactions between valence and arousal in dysphoria which is worth further investigation. These findings may provide novel treatment targets. Instead of focusing solely on symptom reduction, interventions could aim to increase the flexibility of the emotion system. Promising approaches may include cognitive flexibility training to disrupt overly pessimistic prediction loops and mindfulness-based intervention to decouple rigid emotion associations. Furthermore, monitoring network density via digital platforms could serve as an early warning system. At-risk individuals could be identified based on dynamic patterns before severe symptoms emerge. This might pave the way for just-in-time interventions designed to restore adaptive emotional dynamics.

Finally, significant group differences were found in dynamic associations between anticipatory and experienced emotions. Specifically, the predictive effect of anticipatory valence on experienced valence was weaker in dysphoric individuals than in controls. This observation aligns with the biases in affective forecasting that have been consistently documented in depression (4). It may reflect a diminished accuracy in affective forecasting among those with dysphoria. Interestingly, in return, anticipatory valence in dysphoric individuals appears to be more significantly influenced by their prior experienced valence. It supports the reconstructive memory model of future thinking in depression (49) and could be one of possible explanations for the negative bias in anticipatory emotion in depression. Specifically, dysphoric individuals tend to experience more negative emotion and place greater weight on their past experiences when estimating future emotions, which consequently leads to more negative anticipatory emotions. These findings suggest that enhancing the ability of depressed people to flexibly consider situational information when anticipating the future, instead of focusing on negative memories, may be a promising target for improving affective forecasting biases.

Regarding the predictive role of psychophysiological indicators of affective forecasting on depression, we indicated that the dynamics of affective forecasting can predict subsequent depressive symptoms, even when controlling for baseline depression levels. It is worth noting that stronger autoregressions of anticipatory and experienced valence predicted higher levels of depressive symptoms. Emotional inertia, which has been consistently observed in depression (50), is linked to impaired emotion regulation (51). Our results suggest that the inertia of anticipatory and experienced valence may constitute a risk factor for the development of depression. Additionally, we observed that neither individual emotion variables nor the HRV index alone could predict subsequent depressive symptoms. This finding implies that the temporal network may offer more comprehensive information and serve as a more sensitive predictor of depression.

Several limitations should be addressed. Firstly, the fixed-interval ESM schedule may have introduced recall biases due to time lags between experienced events and ESM measurements. Previous research has suggested that although emotional rating bias caused by the delay was similar in depressed and healthy people, delayed answers of healthy individuals showed higher within-person variability (52). Future studies could benefit from employing event-contingent sampling with random intervals to enhance the real-time assessment of emotions linked to specific events. Secondly, the sampling rate was relatively low in current study. Future thinking is highly prevalent in daily life, occurring approximately every 16 minutes during waking hours (10). Further research is warranted to clarify optimal timescale for examining fluctuations in affective forecasting. Thirdly, the correlational nature of our study design limits the ability to draw causal inferences regarding the relationships between affective forecasting dynamics, HRV, and subsequent depressive symptoms. Future research should employ experimental designs (e.g., directly targeting affective forecasting) or more complex longitudinal models, to better elucidate the causal pathways and directionality of these effects. In addition, our sample consisted predominantly of female Chinese college students, which may limit the generalizability of the results. And the exclusion of individuals with high MDQ scores or other psychiatric histories may further restrict the applicability of these findings to broader depressive populations. Future studies should aim to replicate these findings in more diverse samples. It is worth noting that self-monitoring in ESM may introduce reactivity, inflating emotion awareness or reducing ecological validity. Future work should combine passive monitoring with longitudinal sampling to capture more naturalistic emotional dynamics. Finally, the sample size was relatively small. More studies are needed to validate these results in large subclinical and clinical samples.

In conclusion, dysphoric individuals were characterized with more negative anticipatory, experienced and consummatory valence and high consummatory arousal. Most importantly, the temporal network of anticipatory, experienced and consummatory emotions was denser in dysphoric individuals than in controls. Temporal network analysis also indicated that affective forecasting was less accurate and more inflexible in dysphoric individuals compared with controls. Moreover, the dynamic relationship between anticipatory and experienced emotions was a significant predictor of subsequent depression. In addition, we found a significant correlation between reduced HRV and affective forecasting deficit in dysphoria for the first time. Our findings indicate that improving the flexibility of affective forecasting could serve as a potential target for early addressing depression. This study might provide new insights into the psychophysiological mechanisms of affective forecasting deficits in depression and might facilitate early detection and intervention on depression.

## Data Availability

The raw data supporting the conclusions of this article will be made available by the authors, without undue reservation.

## References

[1] Clark A . Whatever next? Predictive brains, situated agents, and the future of cognitive science. Behav Brain Sci. (2013) 36:181–204. doi: 10.1017/S0140525X12000477, PMID: 23663408

[2] Szpunar KK Spreng RN Schacter DL . A taxonomy of prospection: Introducing an organizational framework for future-oriented cognition. Proc Natl Acad Sci United States America. (2014) 111:18414–21. doi: 10.1073/pnas.1417144111, PMID: 25416592 PMC4284580

[3] Beck AT . "The development of depression: A cognitive model." In R. J. Friedman and M. M. Katz, editors. The psychology of depression: Contemporary theory and research. John Wiley & Sons. (1974).

[4] Rizeq J . Affective forecasting and psychopathology: A scoping review. Clin Psychol Rev. (2024) 84:102392. doi: 10.1016/j.cpr.2024.102392, PMID: 38244480

[5] Roepke AM Seligman ME . Depression and prospection. Br J Clin Psychol. (2016) 55:23–48. doi: 10.1111/bjc.12087, PMID: 26096347

[6] Hallford DJ Barry TJ Austin DW Raes F Takano K Klein B . Impairments in episodic future thinking for positive events and anticipatory pleasure in major depression. J Affect Disord. (2020) 260:536–43. doi: 10.1016/j.jad.2019.09.039, PMID: 31539690

[7] Holmes EA Blackwell SE Burnett Heyes S Renner F Raes F . Mental imagery in depression: Phenomenology, potential mechanisms, and treatment implications. Annu Rev Clin Psychol. (2016) 12:249–80. doi: 10.1146/annurev-clinpsy-021815-092925, PMID: 26772205

[8] Wenze SJ Gunthert KC German RE . Biases in affective forecasting and recall in individuals with depression and anxiety symptoms. Pers Soc Psychol Bull. (2012) 38:895–906. doi: 10.1177/0146167212447242, PMID: 22649114

[9] Moustafa AA Morris AN ElHaj M . A review on future episodic thinking in mood and anxiety disorders. Rev Neurosci. (2018) 30:85–94. doi: 10.1515/revneuro-2017-0055, PMID: 29858910

[10] Barsics C van der Linden M D’Argembeau A . Frequency, characteristics, and perceived functions of emotional future thinking in daily life. Q J Exp Psychol. (2016) 69:217–33. doi: 10.1080/17470218.2015.1051560, PMID: 26140455

[11] Myin-Germeys I Oorschot M Collip D Lataster J Delespaul P Van Os J . Experience sampling research in psychopathology: Opening the black box of daily life. Psychol Med. (2009) 39:1533–47. doi: 10.1017/S0033291708004947, PMID: 19215626

[12] Shiffman S Stone AA Hufford MR . Ecological momentary assessment. Annu Rev Clin Psychol. (2008) 4:1–32. doi: 10.1146/annurev.clinpsy.3.022806.091415, PMID: 18509902

[13] Li X Zhang Y-T Huang Z-J Chen X-L Yuan F-H Sun X-J . Diminished anticipatory and consummatory pleasure in dysphoria: Evidence from an experience sampling study. Front Psychol. (2019) 10:2124. doi: 10.3389/fpsyg.2019.02124, PMID: 31607980 PMC6761272

[14] Wu H Mata J Furman DJ Whitmer AJ Gotlib IH Thompson RJ . Anticipatory and consummatory pleasure and displeasure in major depressive disorder:An experience sampling study. J Abnormal Psychol. (2017) 126:149. doi: 10.1037/abn0000244, PMID: 27936838 PMC5305427

[15] Mathersul DC Ruscio AM . Forecasting the future, remembering the past: Misrepresentations of daily emotional experience in generalized anxiety disorder and major depressive disorder. Cogn Ther Res. (2020) 44:73–88. doi: 10.1007/s10608-019-10048-5

[16] Zetsche U Bürkner P-C Renneberg B . Future expectations in clinical depression:Biased or realistic? J Abnormal Psychol. (2019) 128:678. doi: 10.1037/abn0000452, PMID: 31403805

[17] Buehler R McFarland C Spyropoulos V Lam KCH . Motivated prediction of future feelings: Effects of negative mood and mood orientation on affective forecasts. Pers Soc Psychol Bull. (2007) 33:1265–78. doi: 10.1177/0146167207303014, PMID: 17586732

[18] Hepburn SR Barnhofer T Williams JMG . Effects of mood on how future events are generated and perceived. Pers Individ Dif. (2006) 41:801–11. doi: 10.1016/j.paid.2006.03.022

[19] Gullett N Zajkowska Z Walsh A Harper R Mondelli V . Heart rate variability (HRV) as a way to understand associations between the autonomic nervous system (ANS) and affective states: A critical review of the literature. Int J Psychophysiol. (2023) 192:5–42. doi: 10.1016/j.ijpsycho.2023.08.001, PMID: 37543289

[20] Appelhans BM Luecken LJ . Heart rate variability as an index of regulated emotional responding. Rev Gen Psychol. (2006) 10:229–40. doi: 10.1037/1089-2680.10.3.229

[21] Beck AT Steer RA Brown G . Beck depression inventory–II. Psychol Assess. (1996) 8:490–8.

[22] Wang Y-P Gorenstein C . Psychometric properties of the Beck Depression Inventory-II: A comprehensive review. Braz J Psychiatry. (2013) 35:416–31. doi: 10.1590/1516-4446-2012-1048, PMID: 24402217

[23] Hirschfeld RM . The Mood Disorder Questionnaire: A simple, patient-rated screening instrument for bipolar disorder. Primary Care Companion J Clin Psychiatry. (2002) 4:9. doi: 10.4088/PCC.v04n0104, PMID: 15014728 PMC314375

[24] Yang HC Yuan CM Liu TB Li LJ Peng HJ Rong H . Validity of the Chinese version Mood Disorder Questionnaire(MDQ)and the optimal cutoff screening bipolar disorders. Psychiatry Res. (2011) 189:446–50. doi: 10.1016/j.psychres.2011.02.007, PMID: 21402414

[25] Wang Y-Y Xu D-D Liu R Yang Y Grover S Ungvari GS . Comparison of the screening ability between the 32-item Hypomania Checklist(HCL-32)and the Mood Disorder Questionnaire (MDQ) for bipolar disorder: A meta-analysis and systematic review. Psychiatry Res. (2019) 273:461–6. doi: 10.1016/j.psychres.2019.01.061, PMID: 30684793

[26] Quigley L Dobson KS . An examination of trait, spontaneous and instructed emotion regulation in dysphoria. Cogn Emotion. (2014) 28:622–35. doi: 10.1080/02699931.2013.848786, PMID: 24188287

[27] Green P MacLeod CJ . SIMR: An R package for power analysis of generalized linear mixed models by simulation. Methods Ecol Evol. (2016) 7:493–8. doi: 10.1111/2041-210X.12504

[28] Kroenke K Spitzer RL Williams JB . The PHQ-9: Validity of a brief depression severity measure. J Gen Internal Med. (2001) 16:606–13. doi: 10.1046/j.1525-1497.2001.016009606.x, PMID: 11556941 PMC1495268

[29] Wang W Bian Q Zhao Y Li X Wang W Du J . Reliability and validity of the Chinese version of the Patient Health Questionnaire (PHQ-9) in the general population. Gen Hosp Psychiatry. (2014) 36:539–44. doi: 10.1016/j.genhosppsych.2014.05.021, PMID: 25023953

[30] Chan RC Shi YF Lai MK Wang YN Wang Y Kring AM . The Temporal Experience of Pleasure Scale (TEPS): Exploration and confirmation of factor structure in a healthy Chinese sample. PLoS One. (2012) 7:e35352. doi: 10.1371/journal.pone.0035352, PMID: 22530007 PMC3329425

[31] Gard DE Gard MG Kring AM John OP . Anticipatory and consummatory components of the experience of pleasure: A scale development study. J Res Pers. (2006) 40:1086–102. doi: 10.1016/j.jrp.2005.11.001

[32] Yang ZY Zheng YC Yang X Wang YT Feng ZZ . The development of the Negative Bias in Prospection Scale: A novel assessment of dysfunctional prospection in depression. Psych J. (2023) 12:84–91. doi: 10.1002/pchj.590, PMID: 36116919

[33] Bringmann LF Pe ML Vissers N Ceulemans E Borsboom D Vanpaemel W . Assessing temporal emotion dynamics using networks. Assessment. (2016) 23(4):425–35. 10.1177/1073191116645909 27141038

[34] Li F Liu G Zou Z Yan Y Huang X Liu X . A classification framework for depressive episode using RR intervals from smartwatch. IEEE Trans Affect Comput. (2023) 15(3):1387–99.

[35] Orphanidou C Bonnici T Charlton P Clifton D Vallance D Tarassenko L . Signal-quality indices for the electrocardiogram and photoplethysmogram: Derivation and applications to wireless monitoring. IEEE J Biomed Health Inf. (2014) 19:832–8. doi: 10.1109/JBHI.2014.2338351, PMID: 25069129

[36] Stange JP Li J Xu EP Ye Z Zapetis SL Phanord CS . Autonomic complexity dynamically indexes affect regulation in everyday life. J Psychopathol Clin Sci. (2023) 132:847. doi: 10.1037/abn0000849, PMID: 37410429 PMC10592626

[37] R Core Team . R: A language and environment for statistical computing. Vienna, Austria: R Foundation for Statistical Computing. (2016). Available online at: http://www.R-project.org/.

[38] Isvoranu AM Epskamp S . Which estimation method to choose in network psychometrics? Deriving guidelines for applied researchers. Psychological Methods. (2023) 28(4):925., PMID: 34843277 10.1037/met0000439

[39] Epskamp S van Borkulo CD van der Veen DC Servaas MN Isvoranu AM Riese H . Personalized network modeling in psychopathology: The importance of contemporaneous and temporal connections. Clin Psychol Sci. (2018) 6(3):416–27., PMID: 29805918 10.1177/2167702617744325 PMC5952299

[40] Wenzler S Hagen M Tarvainen MP Hilke M Ghirmai N Huthmacher A-C . Intensified emotion perception in depression:Differences in physiological arousal and subjective perceptions. Psychiatry Res. (2017) 253:303–10. doi: 10.1016/j.psychres.2017.03.040, PMID: 28412613

[41] Ulke C Wittekind DA Spada J Franik K Jawinski P Hensch T . Brain arousal regulation in SSRI-medicated patients with major depression. J Psychiatr Res. (2019) 108:34–9. doi: 10.1016/j.jpsychires.2018.11.003, PMID: 30448695

[42] Moretta T Benvenuti SM . Early indicators of vulnerability to depression: The role of rumination and heart rate variability. J Affect Disord. (2022) 312:217–24. doi: 10.1016/j.jad.2022.06.049, PMID: 35760196

[43] Kemp AH Quintana DS Gray MA Felmingham KL Brown K Gatt JM . Impact of depression and antidepressant treatment on heart rate variability: A review and meta-analysis. Biol Psychiatry. (2010) 67:1067–74. doi: 10.1016/j.biopsych.2009.12.012, PMID: 20138254

[44] Koch C Wilhelm M Salzmann S Rief W Euteneuer F . A meta-analysis of heart rate variability in major depression. psychol Med. (2019) 49:1948–57. doi: 10.1017/S0033291719001351, PMID: 31239003

[45] Castellano P Gigli V Ghezzi V Ang Y-S Schettino M Pizzagalli DA . Momentary gustative-olfactory sensitivity and tonic heart rate variability are independently associated with motivational behavior. Int J Psychophysiol. (2023) 186:1–9. doi: 10.1016/j.ijpsycho.2023.01.010, PMID: 36738932

[46] Mather M Yoo HJ Clewett DV Lee TH Greening SG Ponzio A . Higher locus coeruleus MRI contrast is associated with lower parasympathetic influence over heart rate variability. Neuroimage. (2017) 150:329–35. doi: 10.1016/j.neuroimage.2017.02.025, PMID: 28215623 PMC5391255

[47] Shin KE Newman MG Jacobson NC . Emotion network density is a potential clinical marker for anxiety and depression: Comparison of ecological momentary assessment and daily diary. Br J Clin Psychol. (2022) 61:31–50. doi: 10.1111/bjc.12295, PMID: 33963538 PMC8572316

[48] Petrolini V Viola M . Core affect dynamics: Arousal as a modulator of valence. Rev Philosophy Psychol. (2020) 11:783–801. doi: 10.1007/s13164-020-00474-w

[49] Miloyan B Pachana NA Suddendorf T . The future is here: A review of foresight systems in anxiety and depression. Cogn Emot. (2014) 28(5):795–810., PMID: 24320101 10.1080/02699931.2013.863179

[50] Brose A Schmiedek F Koval P Kuppens P . Emotional inertia contributes to depressive symptoms beyond perseverative thinking. Cogn Emot. (2015) 29(3):527–38., PMID: 24820350 10.1080/02699931.2014.916252

[51] Chen T Wang P Wang Y Irish M . Combining experience sampling with temporal network analysis to understand inertia of negative emotion in dysphoria. J Affect Disord. (2023) 338:246–53., PMID: 37315591 10.1016/j.jad.2023.06.006

[52] Eisele G Vachon H Myin-Germeys I Viechtbauer W . Reported affect changes as a function of response delay: findings from a pooled dataset of nine experience sampling studies. Front Psychol. (2021) 12:580684. doi: 10.3389/fpsyg.2021.580684, PMID: 33716852 PMC7952513

