# Supplementary material for: Affective forecasting dynamics as an early intervention target in depression: evidence from ecological monitoring and temporal network analysis
Source: Front Psychiatry. 2026 Jan 12;16:1739976. doi: 10.3389/fpsyt.2025.1739976 (PMC12832834; doi:10.3389/fpsyt.2025.1739976)

**Table S1.**

Behavioral factors included in ESM surveys

| **Variable** | **Item** | **Rating** |
| --- | --- | --- |
| Body posture | During the past 10 minutes, were you mostly: lying down; sitting; standing; walking? | 1 (lying down), 2 (sitting), 3 (standing), 4 (walking) |
| Social interaction | During the past 10 minutes, were you mostly: alone; with other people, but without interaction; interacting with other people? | 1 (alone), 2 (with other people, but without interaction), 3 (interacting with other people) |
| Eating | In the past 30 minutes, have you had something to eat? | 1 (yes), 2 (no) |
| Drinking | In the past 30 minutes, have you had something to drink? | 1 (yes), 2 (no) |
| Physical activity | In the past 30 minutes, have you been physically active? | 1 (yes), 2 (no) |

**Table S2.**

Group differences in affective forecasting (comparisons of edges between anticipatory and experienced emotions)

|  | Dysphoric group (*N* = 38) | Control group  (*N* = 53) | t/χ^2^ | *p* |
| --- | --- | --- | --- | --- |
|  | (*M* ± *SD*) | (*M* ± *SD*) |  |  |
| Anti_val-Anti_val | 0.07±0.09 | 0.09±0.09 | 0.80 | 0.426 |
| **Anti_val-Anti_ar** | **0.07±0.11** | **-0.01±0.05** | **-4.16** | **p < 0.001*** |
| **Anti_val-Exp_val** | **0.09±0.06** | **0.16±0.14** | **3.27** | **0.002*** |
| Anti_val-Exp_ar | 0.03±0.05 | -0.01±0.07 | -2.58 | 0.01 |
| **Anti_ar-Anti_val** | **0.12±0.07** | **0.03±0.01** | **-8.05** | **p < 0.001*** |
| Anti_ar-Anti_ar | 0.11±0.06 | 0.11±0.08 | 0.015 | 0.998 |
| Anti_ar-Exp_val | 0.05±0.09 | 0.07±0.06 | 1.704 | 0.092 |
| Anti_ar-Exp_ar | 0.11±0.02 | 0.15±0.00 | 1.419 | 0.164 |
| **Exp_val-Anti_val** | **0.10±0.05** | **0.03±0.05** | **-7.75** | **p < 0.001*** |
| **Exp_val-Anti_ar** | **0.09±0.04** | **0.02±0.08** | **-6.50** | **p < 0.001*** |
| **Exp_val-Exp_val** | **0.11±0.08** | **0.06±0.03** | **-4.284** | **p < 0.001*** |
| Exp_val-Exp_ar | 0.05±0.04 | 0.04±0.05 | -0.075 | 0.940 |
| Exp_ar-Anti_val | -0.01±0.01 | 0.00±0.02 | 1.22 | 0.146 |
| **Exp_ar-Anti_ar** | **-0.02±0.00** | **0.06±0.07** | **8.75** | **p < 0.001*** |
| **Exp_ar-Exp_val** | **-0.01±0.02** | **0.01±0.03** | **4.75** | **p < 0.001*** |
| **Exp_ar-Exp_ar** | **-0.01±0.00** | **-0.03±0.04** | **-3.426** | **0.001*** |

*Note.* *significant after the the Bonferroni correction; Anti, Anticipatory; Exp, Experienced; val, valence; ar, arousal.

**Fig. S1.** Temporal networks of anticipatory, experienced and consummatory emotions in non-dysphoric (A) and dysphoric (B) individuals in the sensitivity analysis including overnight lags. The green lines represent positive partial correlations, while red lines represent negative ones. Thicker edges represent stronger associations between nodes. Only significant edges were retained (p < 0.05). A_V: Anticipatory_Valence; E_V: Experienced_Valence; C_V: Consummatory_Valence; A_A: Anticipatory_Arousal; E_A: Experienced_Arousal; C_A: Consummatory_Arousal.





**Fig. S2.** Temporal networks of anticipatory, experienced and consummatory emotions in non-dysphoric (A) and dysphoric (B) individuals in the sensitivity analysis adopting the non-orthogonal method. The green lines represent positive partial correlations. Thicker edges represent stronger associations between nodes. Only significant edges were retained (p < 0.05). A_V: Anticipatory_Valence; E_V: Experienced_Valence; C_V: Consummatory_Valence; A_A: Anticipatory_Arousal; E_A: Experienced_Arousal; C_A: Consummatory_Arousal.


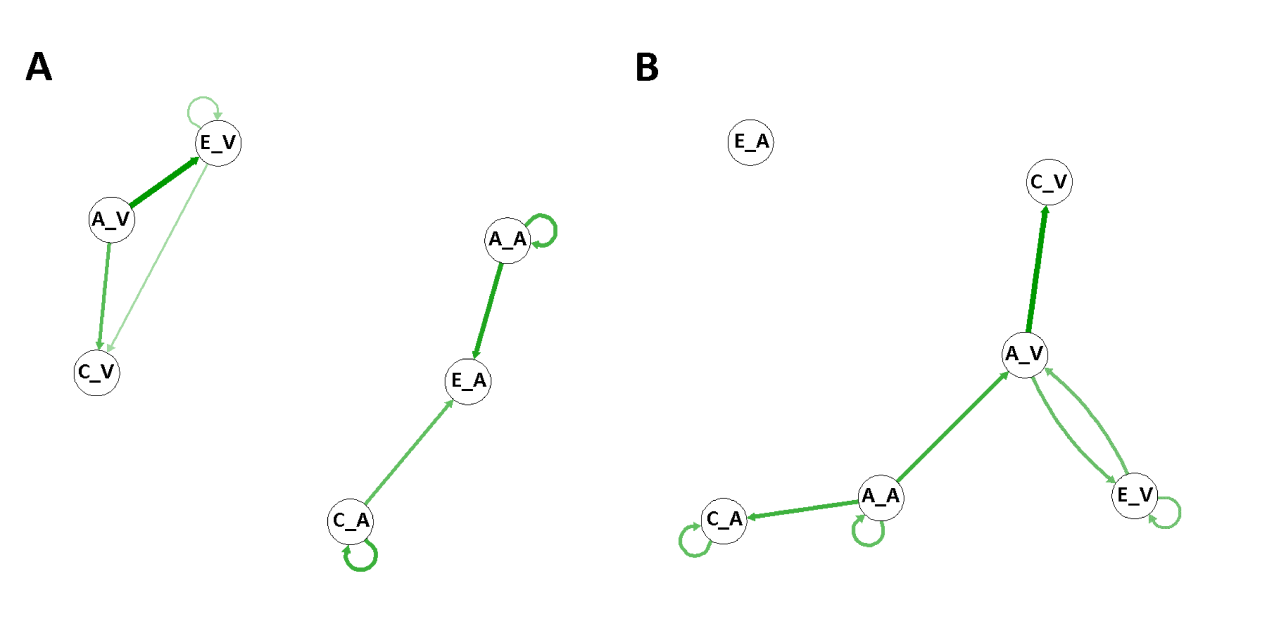

Supplement: Supplementary file 1 [file Table1.docx]
